# Protective Effect of Boric Acid on Oxidative DNA Damage In Chinese Hamster Lung Fibroblast V79 Cell Lines 

**DOI:** 10.22074/cellj.2016.3847

**Published:** 2016-01-17

**Authors:** Sezen Yılmaz, Aylin Ustundag, Ozge Cemiloglu Ulker, Yalcın Duydu

**Affiliations:** Department of Toxicology, Faculty of Pharmacy, Ankara University, Ankara, Turkey

**Keywords:** Boric Acid, Boron, Comet Assay, Oxidative DNA Damage

## Abstract

**Objective:**

Many studies have been published on the antioxidative effects of boric acid (BA) and sodium borates in *in vitro* studies. However, the boron (B) concentrations tested in these *in vitro* studies have not been selected by taking into account the realistic blood B concentrations in humans due to the lack of comprehensive epidemiological studies. The recently published epidemiological studies on B exposure conducted in China and Turkey provided blood B concentrations for both humans in daily life and workers under extreme exposure conditions in occupational setting. The results of these studies have made it possible to test antioxidative effects of BA in *in vitro* studies within the concentra- tion range relevant to humans. The aim of this study was to investigate the protective ef- fects of BA against oxidative DNA damage in V79 (Chinese hamster lung fibroblast) cells. The concentrations of BA tested for its protective effect was selected by taking the blood B concentrations into account reported in previously published epidemiological studies. Therefore, the concentrations of BA tested in this study represent the exposure levels for humans in both daily life and occupational settings.

**Materials and Methods:**

In this experimental study, comet assay and neutral red uptake (NRU) assay methods were used to determinacy to toxicity and genotoxicity of BA and hydrogen peroxide (H_2_O_2_).

**Results:**

The results of the NRU assay showed that BA was not cytotoxic within the tested concentrations (3, 10, 30, 100 and 200 µM). These non-cytotoxic concentrations were used for comet assay. BA pre-treatment significantly reduced (P<0.05, one-way ANOVA) the DNA damaging capacity of H_2_O_2_ at each tested BA concentrations in V79 cells.

**Conclusion:**

Consequently, pre-incubation of V79 cells with BA has significantly reduced the H_2_O_2_-induced oxidative DNA damage in V79 cells. The protective effect of BA against oxidative DNA damage in V79 cells at 5, 10, 50, 100 and 200 μM (54, 108, 540, 1080, and 2161 ng/ml B equivalents) concentrations was proved in this *in vitro* study.

## Introduction

Boron (B) is the fifth element with the symbol "B" in the periodic table. B does not exist as elemental form in the environment, whereas it is generally found as borates, borax, boric acid (BA), colemanite, ulexite, etc. BA and sodium borates are the most widely used B compounds in the industrial, agricultural, and medicinal products (1,3). The previously published studies are consistently pointed out that B is an essential element for plants and beneficial in certain concentrations also for humans (4,5). Accordingly, B supplementation has a beneficial effect on the bone mineral density, brain function, cognitive performance, regulation of the normal inflammatory response, and lipid levels in serum, as well as B can be protective against lipid peroxidation, oxidative stress, DNA damage and prostate cancer by inhibiting the prostate specific antigen (6,15). In spite of these well-known beneficial effects of B in humans, BA and sodium borates are classified as toxic to reproduction and development (Category 1B, H360DF) in the classification, labeling and packaging (CLP) regulation and included into the candidate list of substances of very high concern (16). This classification is mainly based on the results of experimental studies in animals. Accordingly no-observed-adverse-effectslevels (NOAELs) for B mediated toxic effects on the development and reproduction in rats were identified as 9.6 and 17.5 mg/kg/day, respectively (17). 

Turkey possesses the largest B reserves in the world. As a natural consequence of this situation many people living in the south Marmara region around the B deposits and mining areas are exposed to high level of B (18,20). Therefore, the classification of BA and sodium borates in Category 1B (H360DF) has initiated public concern about the potential unfavorable effects of high level of B exposure in the people living in such residential areas. From this point of view, investigating the antioxidative or other beneficial effects of B compounds might be considered to have a lesser value. However, it should be kept in mind that in the CLP regulation of the chemicals are assigned to the hazard categories according to the hazard assessment procedure. It simply means that risk assessment have no value in assigning the chemicals to hazard categories in the CLP regulation. Therefore, certain levels of daily B intake (or exposure) might still be safe and beneficial. Indeed B mediated reprotoxic effects have not been proven in recently published major epidemiological studies conducted in China and Turkey (9,21,23). Both studies have concluded that human B exposures, even in the highest exposure cohorts, are too low to reach the blood (and target tissue) concentrations that would be required to exert adverse effects on reproductive functions (22,24). Moreover, protective effects of B exposure have also been reported on the sperm morphology, sperm motility and DNA integrity in the semen samples of manufacturing workers under the exposure conditions of the BA production plant in B andırma, Turkey (21,23). Consequently, the key parameter which determines the benefit and harm is the daily B intake level. 

The present study aimed to investigate the protective effect of BA on oxidative DNA damage in V79 cells with BA concentrations relevant to humans. The B concentrations tested in this study are based on the blood B concentrations in humans reported in the recently published epidemiological studies in China and Turkey (19, 22). The potential DNA damaging effect of hydrogen peroxide (H2O2) was tested in V79 cells pre-incubated with increasing concentrations (5, 10, 50, 100 and 200 μM) of BA using the alkaline comet assay. The possible cytotoxic effects of BA were identified using the NRU.

## Materials and Methods

This study was conducted in the laboratory of Ankara University Faculty of Pharmacy Department of Pharmaceutical Toxicology in 2013. 

### Chemicals

BA and H_2_O_2_ were purchased from Sigma-Aldrich (Germany). For cell culture, we used Dulbecco’s Modified Eagle’s Medium (DMEM, Biological Industries, Israel) and fetal calf serum (FCS, Sigma- Aldrich, Germany). Dimethyl sulfoxide (DMSO) was the product of Merck (Germany). The NR solution, normal melting point agarose (NMA) and low melting point agarose (LMA) were purchased from Sigma-Aldrich (Germany). Sodium chloride (NaCl), disodium ethylene diaminetetraacetic acid (Na2EDTA), Triton-X 100, Tris and sodium sarcosinate were purchased from Amresco (OH, USA). Ethidium bromide (EtBr) was purchased from Sigma-Aldrich (Germany) for fluorescent dying in the comet assay.

### Setting the pre-treatment concentrations of boric acid

This experimental study is an original article conducted to determine the protective effect of BA against the H_2_O_2_-induced oxidative DNA damage in V79 cells that was tested at concentrations representing the blood B levels in humans. Accordingly the most recent epidemiological studies conducted in China and Turkey was comprehensively reviewed (19-23). The highest mean blood B concentrations reported in China and Turkey for the B exposed workers were 499.2 ± 790.6 ppb (20.4–3568.9) and 223.89 ± 69.49 ng/g (152.82–454.02), respectively, as shown in table 1. The extreme blood B concentrations determined in China surely reflects extreme daily B intake levels accompanied with poor hygienic conditions. Therefore, such a high level of blood B concentration seems not possible in western countries applying the standard risk management regulations in their workplaces. Nevertheless we decided to fix the upper concentration of BA at 200 μM (corresponds to 2163 ppb B) in investigating the protective role of BA against H_2_O_2_- induced DNA damage.

The mean blood B concentrations of the control workers reported in the above mentioned epidemiological studies were taken into consideration in deciding to the lowest test concentration for BA. The mean blood B concentration of the Chinese control group representing the sampling period of 2004 was comparable to the blood B concentration of the control group from the Turkish study (Table 1). 

On the other hand, Yazbeck et al. (25) reported a study on the correlation between B concentrations in drinking water and blood B concentrations in Northern France. According to this study, the mean blood B concentration was 123 ng/g, for the population living in municipalities with water B levels less than 0.3 mg/L. The current drinking water limits for B are 1 mg/L and 2.4 mg/L in the European Union (EU) Drinking Water Directive (98/83/EC) and World Health Organization (WHO) Guidelines for Drinking Water Quality 4th ed. (2011), respectively. Accordingly we decided to set the lowest B concentration to 5 μM (corresponds to 54 ppb B). Thus, the concentration range which we selected to study the protective effect of B against oxidative DNA damage was based on the results of the epidemiological studies. The BA concentrations that we used in this study and the corresponding B equivalents are compiled in table 2.

Cytotoxicity of BA in V79 cells was determined by means of the NRU assay as described previously (26, 27). Briefly, 1×10^4^ cells were plated in 0.2 ml DMEM (with 10% FCS and 1% penicillin/streptomycin) per well in 96-well tissue-culture plates and allowed to attach and grow for 24 hours at 37˚C. BA (3, 10, 30, 100, and 200 μM) were then added to the cell culture medium. After 18 hours, the medium was replaced by fresh medium containing 50 μg/ml NR solution, and the incubation was continued for 3 hours at 37˚C. Thereafter, the medium was withdrawn, and cells were washed two times with phosphate buffered saline (PBS), and fixed with 0.2 mL glacial acetic acid/ water/ethanol (1:49:50, v/v/v) per well; the plates were shaken for 20 minutes to solubilize the NR. Then NR absorbance was measured at 540 nm (SpectraMax, Molecular Devices Inc., USA).

**Table 1:** The blood boron (B) concentrations reported in the epidemiological studies conducted in China and Turkey


Blood B concentrations (ppb) reported in the epidemiological study conducted in China				
Member	Control		Community comparison	Exposed

Xing, 2008 (sampled in 2003)	22.1 ± 6.7 (14.0–33.2)		-	204.8 ± 356.8 (27.1–2003.5)
Xing, 2008 (sampled in 2004)	48.0 ± 23.9 (8.2–113.0)		96.5 ± 90.8 (3.3–536)	499.2 ± 790.6 (20.4–3568.9)
**Blood B concentrations (ng/g) reported in the epidemiological study conducted in Turkey**				
	**Control**	**Low exposure**	**Medium exposure**	**High exposure**
Duydu, 2011	<48.5	72.94 ± 15.43 (48.46–99.91)	121.68 ± 15.62 (100.51–146.07)	223.89 ± 69.49 (152.82–454.02)


Mean ± SD, range in parenthesis. Community comparison are not working in the B industry but living in the B reach area.

**Table 2:** The boric acid concentrations used in pre-treatment of V79 cells


	Boric acid concentrations used in pre-treatment of V79 cells				

H_3_BO_3_(μM)	5	10	50	100	200
H_3_BO_3_, ppb (ng/ml)	309	618	3090	6180	12360
B equivalent, ppb (ng/ml)	54	108	540	1080	2161


Molecular weight of H_3_BO_3_: 61.83 g/mol, atomic weight of B: 10.81 g/mol and conversion factor for equivalent dose of B: 0.1748.

#### Comet assay

The alkaline comet assay was based on the standard method as described earlier (28-30) with minor modifications. Initially, 5×10^4^ V79 cells were seeded into 25 cm^2^ flasks containing DMEM with 10% FCS and cultured for 48 hours at 37˚C. The cells were pre-treated with BA at the concentrations of 5, 10, 50, 100 and 200 μM for 16 hours at 37˚C. Thereafter, the cells were treated with H_2_O_2_ at two concentrations (50 and 100 μM) for 1 hour at 37˚C. Afterwards the cells were harvested in appropriate manner and the cell suspensions (1-2×10^4^ cells/50 μL) were mixed with 100 μL of LMA (0.5%, in PBS, Sigma- Aldrich, Germany). These final cell suspensions were rapidly pipetted onto the pre-coated slides with NMA (1%), allowed to spread using a cover slip, and maintained on an ice-cold flat tray for 5 minutes for solidification.

After removal of the cover slip, the slides were immersed into cold lysing solution (2.5 M NaCl,100 mM Na2EDTA, 10 mM Tris, and 1% sodium sarcosinate at pH=10) containing freshly added 1% Triton-X 100 and 10% DMSO and were left for at least 1 hour at 4˚C. The untreated cells, the cells treated with solely BA, and the cells treated with H_2_O_2_ were not immersed simultaneously into same lysing solution. The slides were removed from the lysing solution, drained, and placed side by side in a horizontal gel electrophoresis tank. The tank was filled with freshly prepared electrophoresis solution (1 mM Na2EDTA and 300 mM NaOH, pH=13). The time of alkali denaturation and electrophoresis (24 V, 300 mA) was 20 minutes each. Afterwards the slides were neutralized with tris buffer (0.4 M Tris, pH=7.5) and allowed to stand for 5 minutes in room temperature (the neutralization step was repeated 3 times). The slides were stained with 65 μL of EtBr (20 μg/mL), covered with a cover slip and analyzed within 3-4 hours. Slides were examined on a fluorescence microscope (Leica DM 1000, Germany) with the Comet Assay IV Software. The Images of 100 randomly selected cells were analyzed for each group. Tail % intensity was used as the measure of the DNA damage in V79 cells.

### Statistical analysis

The SPSS (SPSS Inc., USA) for Windows Release 20.0 was used for all data analysis. The results from the comet assay were expressed as median, and the results of the tail intensities of the control and the treated groups were statistically compared using one-way ANOVA test. Post hoc analysis of group differences was performed by the Fisher’s least significant difference (LSD) test. The limit for statistical significance was fixed as P<0.05. 

## Results

### Cytotoxicity assay

**Fig.1:**
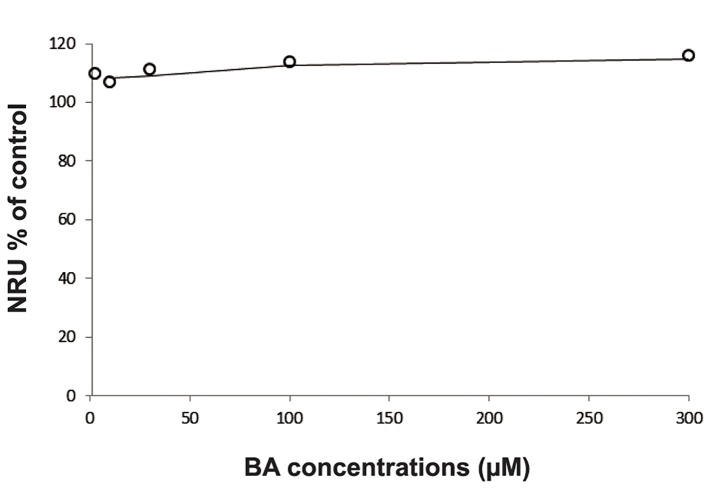
The NRU assay results of BA in V79 cells. NRU; Ndeutral red uptake and BA; Boric acid.

According to the results of the NRU assay BA was not cytotoxic within the tested concentrations (3, 10, 30, 100 and 200 µM). This concentration range covers the BA concentrations ( (5, 10, 50, 100 and 200 µM) tested for its protective effect against H_2_O_2_-mediated DNA damage in V79 cells. It proves that the comet assay was performed at non-cytotoxic concentrations (Fig .1). 

### Comet assay

**Fig.2:**
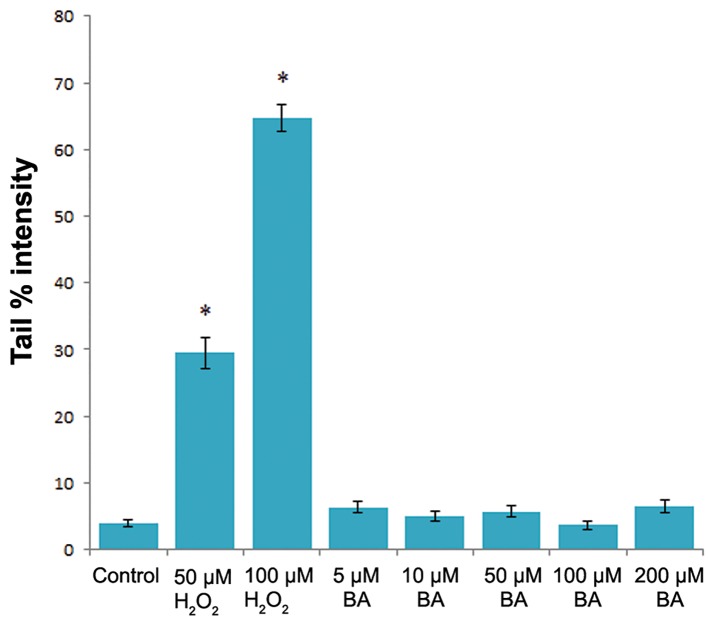
The level of the DNA damge in V79 cells treated with H_2_O_2_ and BA. The "tail % intensity" was used at the measure of the DNA damage. *; Statistically significant (P˂0.05, one-way ANOVA) and BA; Boric acid.

**Fig.3:**
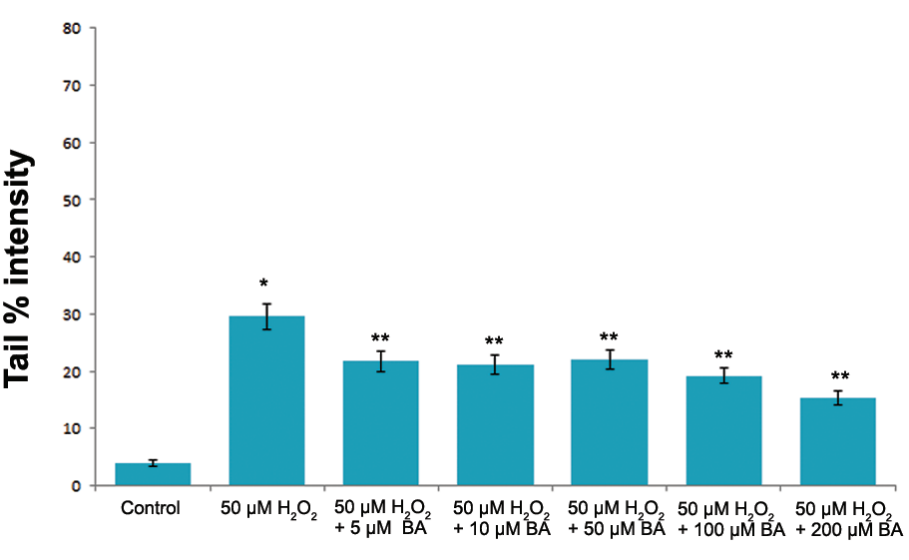
The levels of the DNA damge in V79 cells induced by 50 µM H_2_O_2_. The DNA damage was significantly lower in V79 cells pre-induced with BA. *; Significantly higher than the control (P˂0.05, one-way ANOVA), **; Significantly lower than the DNA damage induced by 50 µM H_2_O_2_(P˂0.05, one-way ANOVA) and BA; Boric acid.

**Fig.4:**
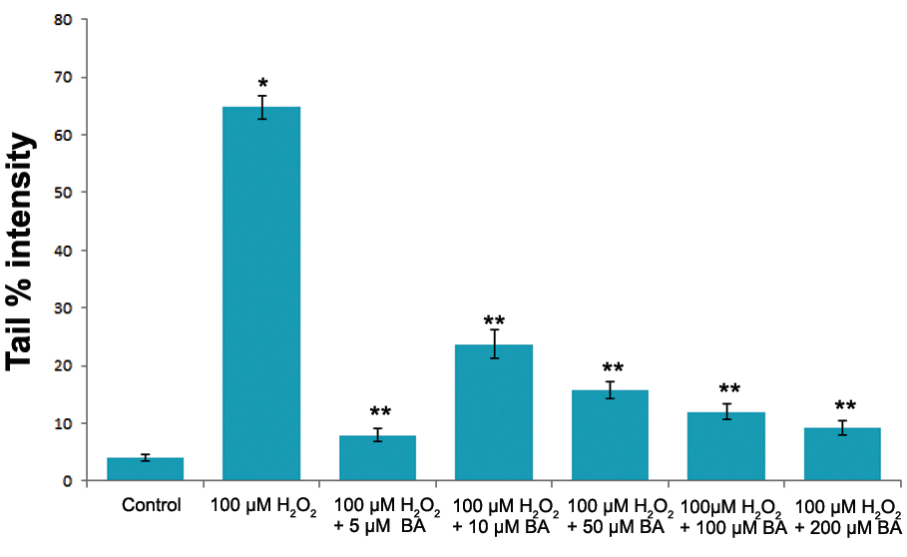
The levels of the DNA damge in V79 cells induced by 100 µM H_2_O_2_. The DNA damage was significantly lower in V79 cells pre-induced with BA. *; Significantly higher than the control (P˂0.05, one-way ANOVA), **; Significantly lower than the DNA damage induced by 100 µM H_2_O_2_(P˂0.05, one-way ANOVA) and BA; Boric acid.

H_2_O_2_ was used as DNA-damaging agent in V79 cells. Both 50 and 100 μM H_2_O_2_ induced statistically significant (P<0.05, one-way ANOVA) DNA damage when compared with the control (Fig .2). The increasing concentrations of BA were also tested for its effects on the DNA integrity of V79 cells. However, statistically significant difference Protective Ef fect of BA on Oxidative DNA Damage in tail % intensity values between control and exposure groups were not determined (P>0.05, one-way ANOVA) as shown in figure 2. On the other hand BA pre-treatment significantly reduced (P<0.05, one-way ANOVA) the DNA damaging capacity of H_2_O_2_ at each tested BA concentrations in V79 cells (Fig .3,4). 

## Discussion

As is known, BA and sodium borates are classified as toxic to reproduction and development in the CLP regulation and included into the candidate list of the substances of very high concern (16). These classifications have raised the public concern about the daily B exposure levels in the population living around the B deposits and mining areas in Turkey. However, these effects have not been proven in recently published comprehensive epidemiological studies conducted in China and Turkey (19,24). 

On the other hand, it should be kept in mind that the chemicals are assigned to the hazard categories according to hazard assessment procedure in the CLP regulation. It simply means that risk assessment have no value in assigning the chemicals to hazard categories. In essence, all chemicals which are toxic to reproduction and development have threshold concentrations to exert their unfavorable effects as it is for BA. Therefore, the daily B intake levels lower than the identified threshold level should be considered as safe and maybe beneficial. Indeed, the available studies show that B is essential for plants and also for some higher animals as frogs and zebrafish (31). Although the studies failed to prove the essentiality of B in humans, numerous beneficial effects of B have been reported in many published studies. B eneficial effects on the strength and trabecular microarchitecture of bone (32), on the human central nervous system (33,34), on the prostate cancer by taking into account the inverse association between dietary B intake and prostate cancer (35,36), on the functions of vitamin D, estrogen, thyroid hormone, insulin, and progesterone (31), on the antioxidant enzyme activities(14), and on reducing the incidence of arthritis (37) are some of the well documented special features of B. Additionally, unfavorable effects of the B deprivation have also been documented in some of the above mentioned studies. 

All of these studies indicate the benefits of B at the dietary intake levels. Nowadays some beneficial effects have also been reported at higher exposure levels in occupational settings. The mean daily B exposure in the high exposure group in B andırma (Turkey) BA production plant was 14.45 ± 6.57 (3.32–35.62) mg/day (19). Although it depends on the use of some personal products and consumed food/water, the daily B intake is considered to be between 1-3 mg/day for humans in daily life (5). When this level of B intake is considered as normal, the daily B exposure in the above mentioned BA production plant might be considered as high. In spite of this high B exposure, some motility and morphology parameters of sperm samples collected from the exposure group were improving with increasing blood B concentrations (mean blood B concentration of high exposure group: 223.89 ± 69.49 ng/g) and the correlation between the dose response was statistically significant. Additionally the oxidative DNA damage in sperm cells was decreasing with increasing blood B concentrations in the same population and this association was also statistically significant (19, 21). These results support a dose dependent increase in the protection capacity of BA against the oxidative DNA damage in sperm cells of the workers employed in B andırma BA production plant. These results encouraged us to prove the protective effect of BA against H_2_O_2_-induced oxidative DNA damage at low and high concentrations reflecting the blood B concentrations of humans in daily life and occupational settings, respectively. 

The lowest and highest BA concentrations used in the pre-incubation period of V79 cells were 5 and 200 µM which are corresponding to 54 and 2161 ng/ml B equivalents, respectively. These concentrations represent the blood B concentrations of control and high exposure workers in epidemiological studies conducted in China and Turkey (19,22). 

The H_2_O_2_-induced oxidative DNA damage at both 50 and 100 µM concentrations were significantly reduced (P>0.05, one-way ANOVA) in V79 cells pre-incubated with 5, 10, 50, 100, and 200 µM (54, 108, 540, 1080, 2161 ng/ml B equivalents) BA concentrations. This result suggests a protective effect of BA against oxidative DNA damage at reasonable B exposure levels for humans in daily life or in occupational setting. 

## Conclusion

Consequently, in spite of the unfavorable effects of B in animal experiments at high doses, the daily B intake levels (at concentrations of lower than the threshold for reproductive and developmental toxicity) have beneficial effects in all tested living organisms including humans. Our study covers the daily B exposure as well as the occupational exposure conditions. The protective effect of BA against oxidative DNA damage has been demonstrated within these common and extreme exposure conditions. From this point of view, our results have supported the earlier studies on the antioxidant capacity of BA. However, further studies are needed to investigate the mechanism of the BA mediated protective effect against oxidative DNA damage. 
